# Supplementary material for: Investigating the Role of Circulating miRNAs as Biomarkers in Colorectal Cancer: An Epidemiological Systematic Review
Source: Biomedicines. 2022 Sep 8;10(9):2224. doi: 10.3390/biomedicines10092224 (PMC9496335; doi:10.3390/biomedicines10092224)
Supplement: Supplementary file 1 [file biomedicines-10-02224-s001.zip › biomedicines-1863494-supplementary.pdf]

PRISMA 2020 Checklist

Section and Topic	Item #	Checklist item	Location where item is reported
**TITLE**			
Title	1	Identify the report as a systematic review.	1
**ABSTRACT**			
Abstract	2	See the PRISMA 2020 for Abstracts checklist.	1
**INTRODUCTION**			
Rationale	3	Describe the rationale for the review in the context of existing knowledge.	2
Objectives	4	Provide an explicit statement of the objective(s) or question(s) the review addresses.	2
**METHODS**			
Eligibility criteria	5	Specify the inclusion and exclusion criteria for the review and how studies were grouped for the syntheses.	2
Information sources	6	Specify all databases, registers, websites, organisations, reference lists and other sources searched or consulted to identify studies. Specify the date when each source was last searched or consulted.	2
Search strategy	7	Present the full search strategies for all databases, registers and websites, including any filters and limits used.	Supplementary-additional file 2
Selection process	8	Specify the methods used to decide whether a study met the inclusion criteria of the review, including how many reviewers screened each record and each report retrieved, whether they worked independently, and if applicable, details of automation tools used in the process.	2-3
Data collection process	9	Specify the methods used to collect data from reports, including how many reviewers collected data from each report, whether they worked independently, any processes for obtaining or confirming data from study investigators, and if applicable, details of automation tools used in the process.	3
Data items	10a	List and define all outcomes for which data were sought. Specify whether all results that were compatible with each outcome domain in each study were sought (e.g. for all measures, time points, analyses), and if not, the methods used to decide which results to collect.	3
	10b	List and define all other variables for which data were sought (e.g. participant and intervention characteristics, funding sources). Describe any assumptions made about any missing or unclear information.	3
Study risk of bias assessment	11	Specify the methods used to assess risk of bias in the included studies, including details of the tool(s) used, how many reviewers assessed each study and whether they worked independently, and if applicable, details of automation tools used in the process.	3
Effect measures	12	Specify for each outcome the effect measure(s) (e.g. risk ratio, mean difference) used in the synthesis or presentation of results.	3
Synthesis methods	13a	Describe the processes used to decide which studies were eligible for each synthesis (e.g. tabulating the study intervention characteristics and comparing against the planned groups for each synthesis (item #5)).	3
	13b	Describe any methods required to prepare the data for presentation or synthesis, such as handling of missing summary statistics, or data conversions.	3
	13c	Describe any methods used to tabulate or visually display results of individual studies and syntheses.	-
	13d	Describe any methods used to synthesize results and provide a rationale for the choice(s). If meta-analysis was performed, describe the model(s), method(s) to identify the presence and extent of statistical heterogeneity, and software package(s) used.	3
	13e	Describe any methods used to explore possible causes of heterogeneity among study results (e.g. subgroup analysis, meta-regression).	3
	13f	Describe any sensitivity analyses conducted to assess robustness of the synthesized results.	-
Reporting bias assessment	14	Describe any methods used to assess risk of bias due to missing results in a synthesis (arising from reporting biases).	3
Certainty	15	Describe any methods used to assess certainty (or confidence) in the body of evidence for an outcome.	3

PRISMA 2020 Checklist

Section and Topic	Item #	Checklist item	Location where item is reported
assessment			
**RESULTS**			
Study selection	16a	Describe the results of the search and selection process, from the number of records identified in the search to the number of studies included in the review, ideally using a flow diagram.	4
	16b	Cite studies that might appear to meet the inclusion criteria, but which were excluded, and explain why they were excluded.	4
Study characteristics	17	Cite each included study and present its characteristics.	4
Risk of bias in studies	18	Present assessments of risk of bias for each included study.	10
Results of individual studies	19	For all outcomes, present, for each study: (a) summary statistics for each group (where appropriate) and (b) an effect estimate and its precision (e.g. confidence/credible interval), ideally using structured tables or plots.	4-10 and supplementary-additional file 3
Results of syntheses	20a	For each synthesis, briefly summarise the characteristics and risk of bias among contributing studies.	10
	20b	Present results of all statistical syntheses conducted. If meta-analysis was done, present for each the summary estimate and its precision (e.g. confidence/credible interval) and measures of statistical heterogeneity. If comparing groups, describe the direction of the effect.	11-12
	20c	Present results of all investigations of possible causes of heterogeneity among study results.	11-12
	20d	Present results of all sensitivity analyses conducted to assess the robustness of the synthesized results.	11-12
Reporting biases	21	Present assessments of risk of bias due to missing results (arising from reporting biases) for each synthesis assessed.	-
Certainty of evidence	22	Present assessments of certainty (or confidence) in the body of evidence for each outcome assessed.	-
**DISCUSSION**			
Discussion	23a	Provide a general interpretation of the results in the context of other evidence.	12-13
	23b	Discuss any limitations of the evidence included in the review.	12-13
	23c	Discuss any limitations of the review processes used.	12-13
	23d	Discuss implications of the results for practice, policy, and future research.	12-13
**OTHER INFORMATION**			
Registration and protocol	24a	Provide registration information for the review, including register name and registration number, or state that the review was not registered.	2
	24b	Indicate where the review protocol can be accessed, or state that a protocol was not prepared.	2
	24c	Describe and explain any amendments to information provided at registration or in the protocol.	-
Support	25	Describe sources of financial or non-financial support for the review, and the role of the funders or sponsors in the review.	14
Competing interests	26	Declare any competing interests of review authors.	14
Availability of data, code and other materials	27	Report which of the following are publicly available and where they can be found: template data collection forms; data extracted from included studies; data used for all analyses; analytic code; any other materials used in the review.	14

PRISMA 2020 Checklist

From: Page MJ, McKenzie JE, Bossuyt PM, Boutron I, Hoffmann TC, Mulrow CD, et al. The PRISMA 2020 statement: an updated guideline for reporting systematic reviews. BMJ 2021;372:n71. doi: 10.1136/bmj.n71

For more information, visit: http://www.prisma-statement.org/

Supplementary material – Additional file 2

Systematic search strategy over PubMED and EMBASE:

1. (circulating

   OR
2. Exosomal)
3. (microRNA[MeSH]

   OR
4. miRNA [MeSH])
5. [(colon [MeSH]

   OR
6. colorectal [MeSH])

   AND
7. cancer)]
8. NOT (cells)
9. NOT (tissue)
10. AND ((English[Filter])
11. AND (Humans[Filter])
12. AND ("2012/01/01"[Date - Publication] : "2022/04/01"[Date - Publication]))

Supplementary Material- Additional file 3

Table S1. AUC, sensitivity and specificity values of miRNAs included in the systematic review

Authors, years	miRNA	Cut-off	AUC	Sens [95%CI]	Spec [95%CI]
Silva, 2021	miR-106a-5p	-	0.627	-	-
	miR-542-5p	-	0.685	-	-
	let-7e-5p	-	0.669	-	-
	miR-28-3p	-	0.680	-	-
	Panel	-	0.716 (0.600–0.832)	59.4 (40.8–75.8)	59.4 (40.8–75.8)
Han, 2021	miR-15b	-	0.86 (0.82, 0.91)	81.33	91.80
	miR-16	-	0.58 (0.51, 0.65)	-	-
	miR-21	-	0.75 (0.69, 0.81)	-	-
	miR-31	-	0.75 (0.68, 0.82	-	-
	Panel miRNA-15, miRNA-21, MiRNA-32	-	-	91.95	97.62
Peng, 2020	miR-30e-3p	-	0.731 (0.654, 0.808)	-	-
	miR-31-5p	-	0.669 (0.586, 0.751)	-	-
	miR-34b-3p	-	0.785 (0.715, 0.855)	-	-
	miR-146a-5p	-	0.739 (0.665, 0.813)	-	-
	miR-148a-3p	-	0.648 (0.559, 0.737)	-	-
	miR-192-5p	-	0.652 (0.569, 0.735)	-	-

	six panel	-	0.932	84.6	86.1
	Panel: miR-30e-3p, miR-146a-5p, miR-148a-3p	-	0.883 (0.831, 0.935)	80.0	78.7
Liu, 2020	miRNA-139-3p	-	0.726 (0.603–0.848)	-	-
Pastor-navarro, 2020	miR-21	-	0.913 (0.848 -0.978)	-	-
Li, 2020	miR-20b-5p	-	0.682 (0.651-0.713)	-	-
	miR-329-3p	-	0.852 (0.828-0.875)	-	-
	miR-374b-5p	-	0.914 (0.895-0.933)	-	-
	miR-503-5p	-	0.734 (0.705-0.763)	-	-
BaderElDin, 2020	let-7c	2.370	0.855 (0.77-0.941)	77.6	96.2
	miR-21	3.343	0.936 (0.884-0.989)	80.7	100
	miR-26a	2.370	0.918 (0.857-0.979)	77.6	96.2
	miR-146a	1.900	0.805 (0.708-0.903)	78.0	74.1
Zhao, 2019	mir-150-5p	−0.0325	0.707 (0.649–0.764)	75.2	58.8
	miR-99b-5p	5.0225	0.628 (0.567–0.689)	32.1	90.8
Sabry, 2018	miR-210	0.512	0.934 (0.873–0.995)	88.6	90.1
	miR-21	0.333	0.973 (0.946–1.00)	91.4	95.0
	miR-126	0.294	0.665 (0.571–0.759)	88.6	50.5

Marcuello, 2019	miR-29a-3p, miR-15b-5p, miR-18a-5p, miR-19a-3p, miR-19b-3p, miR-335-5p	-	0.74 (0.65-0.82)	81	56
Liu, 2019	miR-1290	-	0.88 (0.82-0.95)	76.65	90.23
	miR-320d	-	0.81 (0.72-0.90)	88.83	71.66
	panel	-	0.91 (0.85–0.96)	81.21	90.74
Karimi, 2018	miR-301a	-	0.84		
	miR-23a	-	0.90		
Villanueva, 2018	(miR-19a, miR-19b, miR-15b, miR-29a, miR-335, and miR-18a)	-	0.95 (0.903–0.991)	91	90
Yang, 2018	miR-20a	-	0.676	-	-
	miR-486	-	0.629	-	-
Liu, 2018	miR-27a	-	0.773 (0.669–0.876)	75	77.5
	miR-130a	-	0.742 (0.6331–0.8513)	82.5	62.5
Bilegsaikhan, 2018	miR-338-5p	4.15	0.923 (0.882–0.964)	76.3	92.5
Wikberg, 2018	miR-21	-	0.84	-	-
	miR-18a	-	0.56	-	-

	miR-25	-	0.63	-	-
	miR-22	-	0.55	-	-
Wang, 2017	miR-31	-	0.931 (0.872-0.996)	-	-
	miR-141	-	0.949 (0.904-0.993)	-	-
	miR-224-3p	-	0.857 (0.778-0.927)	-	-
	miR-576-5p	-	0.678 (0.573-0.783)	-	-
	miR-4669	-	0.734 (0.625-0.842)	-	-
Wang W, Qu et 2016	miR-210	1.1476	0.821 (0.778–0.859)	74.6 (69.0–79.7)	73.5 (63.9–79.7)
Wang et al, 2017	miR-125a-3p	-	0.6849 (0.5593–0.8025)	-	-
	miR-320c	-	0.5982 (0.4705–0.7259)	-	-
Ng, 2017	miR-139-3p	−3.524	0.993 (0.986- 1.0)	96.6	97.8
Pan, 2017	miR-15b	0.527	0.624(0.535- 0.712)	50.00(38.61- 61.39)	68.75(57.41- 78.65)
	miR-17	0.971	0.660(0.576- 0.745)	67.50(56.11- 77.55)	62.50(50.96- 73.08)
	miR-21	0.686	0.649(0.563- 0.735)	71.25(60.05- 80.82)	52.50(41.02- 63.79)
	miR-26b	0.530	0.708(0.627- 0.789)	72.50(61.38- 81.90)	56.25(44.70- 67.32)
	miR-145	0.840	0.629(0.542- 0.716)	62.50(50.96- 73.08)	61.25(49.70- 71.94)
Bastaminejad, 2017	miR-21	1.1	0.783	86.05	72.97
Sazanov, 2016	miR-21	-	-	65	85
Zekri, 2016	miR-223	-	0. 838 (0.627-1.00)	-	-

	miR-17	-	0.813 (0.589-1.00	-	-
	miR-19a	-	0.825 (0.611-1.00)	-	-
	miR-20a	-	0.788 (0.558-1.00)	-	-
Vychytilova-Faltejskova, 2016	miR-142-5p	-	0.8150	-	-
	miR-23a-3p	-	0.8908	-	-
	miR-27a-3p	-	0.6973	-	-
	miR-376c-3p	-	0.6542	-	-
	Panel	-	0.922	88.7	81.0
Chen, 2016	miR-21	-	-	-	-
	miR-152	-	0.539	-	-
Sarlinova, 2016	miR-21	-	0.7401	71.8	67.5
	miR-221	-	0.7535	71.8	68.7
	miR-150	-	0.6315	57.7	56.2
	panel	-	0.818	80	74
Basati, 2015	miR-194	1.08	0.85 (0.79-0.93)	72	80
	miR-29b	0.66	0.87 (0.80-0.96)	77	75
Yamada, 2015	miR-21	-	0.706 (0.635– 0.770)	-	-
	miR-29a	-	0.741 (0.673–0.802)	-	-
	miR-125b	-	0.806 (0.742– 0.860)	-	-
Nonaka, 2015	miR-103	0.00081	0.662	55.9	75

	miR-720	0.0016	0.630	58.3	56.3
	miR-21	0.0107	0.675	54.7	84.4
Ghanbari, 2014	miR-142-3p	-	0.71	-	-
	miR-26a-5p	-	0.67	-	-
Fang, 2015	miR-24	−1.731	0.839 (0.787-0.892)	78.38	83.85
	miR-320a	−1.006	0.886 (0.845-0.926)	92.79	73.08
	miR-423-5p	−0.854	0.833 (0.780-0.887)	91.89	70.77
Chen, 2015	miR-106a	2.03	0.605 (0.522-0.688)	74.0	44.4
	miR-20a	2.44	0.590 (0.507-0.674)	46.0	73.42
Li, 2015	miR-29b	2.86	0.743	61.4	72.5
Wang, 2014	Panel: miR-21, Let-7g, miR-31, miR-92, miR-181b, miR-203	-9.595	0.923 (0.869–0.976)	96.4	88.1
Nonaka, 2014	miR-199a-3p	0.0010	0.644	47.6	75.0
	miR-21	0.0107	0.675	54.7	84.4
Basati, 2014	miR-21	1.49	0.87 (0.78–0.95)	77	78
Giraldez, 2013	miR19b	0.6538	0.838 (0.7016-0.9746)	81	80
	miR15b	0.5775	0.864 (0.7507-0.9778)	81	80
	miR29a	0.5449	0.866 (0.74-0.98)	81	85

	miR335	0.5494	0.83 (0.697-0.965)	81	85
Luo, 2013	Panel: miR-29a, -106b, -133a, -342-3p, - 532-3p- miR-18a, -20a, -21, -92a, -143, -145, -181b		0.803 (0.774–0.888)		
Wang, 2012	miR-601	-11.50	0.747	69.2	72.4
	miR-760	−8.09	0.788	80	72.4
	Panel: miR-29a, miR-92a and miR-760		0.943 (0.908–0.979)	83.3	93.1
Wang, 2012	miR-21	3.59	0.85	87.5	74.4

Table S2. Fold change values for miRNAs included in the systematic review

Authors	miRNA	Fold change	P-value
Silva, 2021	miR-106a-5p	1.50	0.003
	miR-542-5p	1.79	0.022
	let-7e-5p	1.60	0.011
	miR-28-3p	1.65	0.021
BaderElDin, 2020	let-7c	5.3	< 0.001
	miR-21	8.8	< 0.0001

	miR-26a	5.5	< 0.0001
	miR-146a	3.6	< 0.001
Zhao, 2019	mir-150-5p	−2.6076	< 0.0001
	miR-99b-5p	−2.2347	< 0.001
Karimi, 2018	miR-301a	3.1	< 0.0001
	miR-23a	3.8	< 0.0001
	miR-301a	3.1	< 0.0001
Yang, 2018	miR-20a	0.697	0.01
	miR-486	0.696	0.05
Bilegsaikhan2018	miR-338-5p	4.94	<0.001
Yan, 2017	miR-638	2.930	-
	miR-5787	2.679	-
	miR-8075	2.181	-
	miR-6869-5p	1.948	-
	miR-548c-5p	1.570	-
	miR-486-5p	1.639	-
	miR-3180-5p	1.512	-
Zhang, 2017	miR-4463	6.57	0.005
	miR-5704	2.51	0.0167
	miR-371b-3p	0.48	0.0125

	miR-1247-5p	0.27	0.0152
	miR-1293	0.31	0.0263
	miR-548at-5p	0.36	0.0153
	miR-107	0.40	0.0476
	miR-139-3p	0.14	0.0088
Pan, 2017	miR-15b	2.659	0.0071
	miR-17	1.767	0.0005
	miR-21	2.802	0.0011
	miR-26b	2.796	<0.0001
	miR-145	2.488	0.0047
Zekri, 2016	miR-223	4.06	0.04
	miR-17	2.1	0.03
	miR-19a	2.8	0.021
	miR-20a	2.2	0.04
Vychytilova-Faltejskova, 2016	miR-142-5p	2.63	<0.0001
	miR-23a-3p	2.30	<0.0001
	miR-27a-3p	1.48	<0.0001
	miR-376c-3p	1.39	<0.0001
Chen, 2016	miR-21	1.23	0.28
	miR-152	2.03	0.02

Sarlinova, 2016	miR-21	2.0279	<0.0001
	miR-221	2.3675	<0.0001
	miR-150	0.7027	0.005
Basati, 2015	miR-194	0.88 (0.68–1.02)	< 0.0001
	miR-29b	0.54 (0.45–0.64)	< 0.0001
Ghanbari, 2014	miR-142-3p	11.31	0.029
	miR-26a-5p	10.97	0.023
Wang, 2014	miR-21	4.65	<0.0001
	Let-7g	5.03	<0.0001
	miR-31	0.17	<0.0001
	miR-92a	0.11	<0.0001
	miR-181b	0.33	<0.0001
	miR-203	0.13	<0.0001
Nonaka, 2014	miR-199a-3p	6.82	0.0015
Giraldez, 2013	miR19b	3.03	0.0035
	miR15b	3.21	0.0019
	miR29a	4.11	0.0237

	miR335	4.16	0.0268
Wang, 2012	miR-601	0.09	-
	miR-760	0.04	-

Table S3. Relative expression of miRNAs included in the systematic review

First author, year	miRNA	Relative expression			
		**miRNA reference**	**Median Ct Value Cases (range/SD)**	**Median Ct Value Controls (range/SD)**	**P-value**
Han, 2021	miR-15b	U6	1.21 (0.96, 1.49)	3.94 (3.47, 4.19)	<0.05
	miR-16	U6	0.79 (0.66, 0.94)	0.83 (0.72, 1.01)	>0.05
	miR-21	U6	1.27 (1.12, 1.38)	3.59 (1.25, 4.02)	<0.05
	miR-31	U6	1.22 (1.08, 1.48)	2.16 (0.98, 2.23)	<0.05
Sabry, 2018	miR-210	RNU6B	0.29 ± 0.14	1.18± 0.68	< 0.001
	miR-21	RNU6B	0.21 ± 0.08	0.82± 0.44	< 0.001
	miR-126	RNU6B	0.33 ± 0.19	0.22± 0.11	< 0.004
Wikberg, 2018	miR-21	*C.elegans*miR-39	-6.8	-8.0	<0.001
	miR-18a	*C.elegans*miR-39	-9.8	-10.2	0.003
	miR-25	*C.elegans*miR-39	-8.5	-8.9	<0.001
	miR-22	*C.elegans*miR-39	-10.2	-9.8	0.003
Wang, 2017	miR-125a-3p	miR-30e-5p	8.99687	9.13797	0.003511

	miR-320c	miR-30e-5p	8.28988	8.53069	0.007102
Ng,2017	miR-139-3p	U6	−6.500	-0.628	<0.001
Sarlinova, 2016	miR-21	RNU48	0.25 (0.16-0.47)	0.13 (0.06-0.21)	<0.0001
	miR-221	RNU48	0.04 (0.02-0.06)	0.02 (0.01-0.03)	<0.0001
	miR-150	RNU48	1.98 (0.64-4.35)	2.82 (1.89-5.46)	0.0054
Wang, 2014	miR-21	miR-16	1.39 ± 1.26	6.46 ± 3.12	<0.0001
	Let-7g	miR-16	0.98 ± 0.76	4.93 ± 7.44	<0.0001
	miR-31	miR-16	1.41 ± 2.24	0.24 ± 0.36	<0.0001
	miR-92a	miR-16	1.74 ± 2.04	0.20 ± 0.30	<0.0001
	miR-181b	miR-16	1.75 ± 2.66	0.57 ± 0.83	<0.0001
	miR-203	miR-16	1.45 ± 2.13	0.19 ± 0.33	<0.0001
Basati, 2014	miR-21	RNU6B	1.16 (1.03–2.30)	3.61 (1.53–6.09)	0.0001
Wang, 2012	miR-21	miR-16	3.05	6.10	< 0.001
